# Association of interleukin - 18 gene polymorphism with susceptibility or protective effect to HTLV-1 infection

**DOI:** 10.1186/1742-4690-8-S1-A118

**Published:** 2011-06-06

**Authors:** Virgínia M D Wagatsuma, Daiani C Cilião-Alves, Maurício C Rocha–Junior, Rodrigo Haddad, Oswaldo M Takayanagui, Eduardo A Donadi, Dimas T Covas, Simone Kashima

**Affiliations:** 1 Regional Blood Center of Ribeirão Preto, Ribeirão Preto, Brazil; 2 Faculty of Medicine of Ribeirão Preto, University of São Paulo, Ribeirão Preto, Brazil; 3 Faculty of Pharmaceutical Sciences of Ribeirão Preto, University of São Paulo, Ribeirão Preto, Brazil

## Background

Polymorphisms in promoter region of IL-18 genes have been studied in many chronic inflammatory and infectious disorders. For instance, the IL-18 -137 GG and -607CC polymorphisms have been associated with elevated expression of IL-18, which could contribute for the inflammatory process and favor the antiviral effects of this cytokine. In this study, we evaluated the IL-18 promoter region -137C/G and -607A/C polymorphisms in HTLV-infected patients exhibiting (HAM) or not (HAC) symptomatic disease and in healthy controls.

## Methods

The promoter region polymorphic sites (-137C/G and -607A/C) were evaluated by polymerase chain reaction - sequence specific primers (PCR-SSP) analysis using peripheral blood DNA obtained from HAC (54), HAM (44) and healthy control (150) individuals. Proviral load of infected patients (HAC and HAM) was determined by real-time PCR.

## Results

The -607CC genotype was less frequent for HAC group (p=0.0501; OR=0.4890; CI=0.2480 to 0.9643), and infected patients (p=0.0232; OR=0.5207; CI=0.3033 to 0.8937) compared to healthy controls. The -607AC genotype was more frequent for HAC group (p=0.0387; OR=1.991; CI=1.043 to 3.801), and infected patients (p=0.0376; OR=1.757; CI=1.047 to 2.948) compared to healthy controls. No significant difference was observed for allelic and genotypic frequencies of the -137C/G among deferments groups. No significant difference was observed for allelic and genotypic frequencies of the -137C/G and -607A/C polymorphisms when correlated with proviral load.

## Conclusion

The -607CC (high producer of IL-18) genotype exhibited protective effect against the infection, whereas the -607AC genotype conferred susceptibility to HTLV-1 infection.

